# Cell-based immune anticipation of the omicron variant in SARS-CoV-2 triple-vaccinated cancer patients

**DOI:** 10.1016/j.isci.2025.113727

**Published:** 2025-10-09

**Authors:** Mario Mairhofer, Lea Kausche, Sabine Kaltenbrunner, Maria Pammer, Riad Ghanem, Maike Stegemann, Clemens A. Schmitt

**Affiliations:** 1 Medical Department of Hematology and Oncology at the Kepler University Hospital, Johannes Kepler University, Linz, Upper Austria, Austria; 2 Medical Department of Hematology, Oncology and Tumor Immunology, and the Molecular Cancer Research Center, Charité - Universitätsmedizin Berlin, Germany; 3 Max-Delbrück-Center for Molecular Medicine in the Helmholtz Association, Robert-Rössle-Straße 10, 13125 Berlin, Germany

**Keywords:** health sciences, medicine, medical specialty, immunology, oncology

## Abstract

SARS-CoV-2 infections affect healthcare systems worldwide. Patients with cancer, a particularly vulnerable group, and oncology care takers were offered early access to mRNA-based vaccinations. We report the dynamics of humoral and cellular immune response parameters of 74 patients with cancer and 12 control participants after two basal vaccinations and a booster six months later. Upon booster vaccination, 78% of patients with tumor under active therapy (versus 50.8% prior to the boost) exhibited humoral and cellular spike protein responses, as compared to 100% and 73.3%, respectively, in those without active therapy. Conducted prior to the emergence of the Omicron variant of concern, we found Wuhan-Hu-1 spike-encoding mRNA vaccination to evoke T cell responses against peptides outside and within the Omicron-mutated region of the spike protein. The vast majority of patients with cancer achieved significant antibody titers upon repeated vaccinations. Accordingly, patients with tumor appeared well-protected, indicated by asymptomatic or mild breakthrough infections during the Omicron wave.

## Highlights

• Cellular and humoral immune response dynamics in vaccinated patients with cancer
• Non-spike directed T cell responses as indicators of virus exposure/hybrid immunity
• Preserved cellular reactivity in the anticipation of the Omicron variant
• Effective protection of patients from severe COVID-19 by repeated mRNA vaccinations

## Graphical abstract

**Figure:**
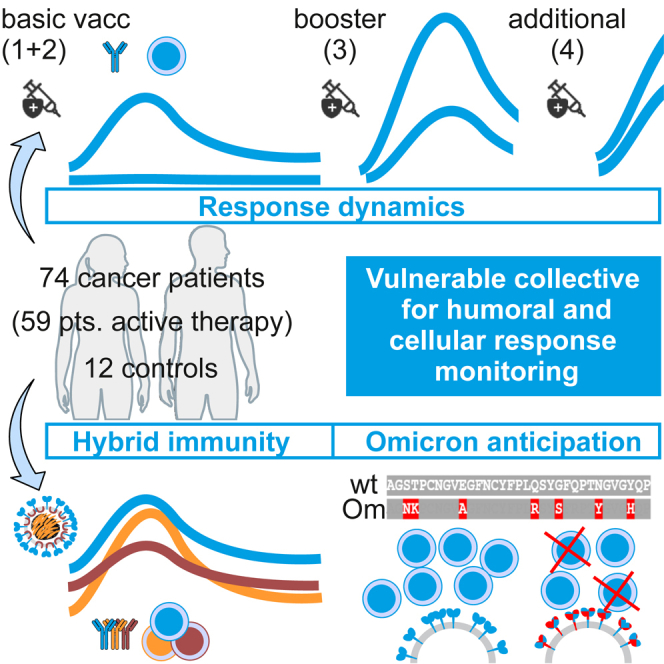

## Introduction

SARS-CoV-2 infections impose a particular risk of morbidity and mortality on patients with cancer.^1^^,^^2^ While mRNA-based spike protein-directed vaccines such as BNT162b2 (Pfizer/BioNTech) and mRNA-1273 (Moderna) significantly lowered the risk of severe COVID-19,^3^^,^^4^ we and others reported responses to a basal immunization with these vaccines to be less profound in patients with tumor (T), especially in patients with hematologic malignancies and exposure to CD20 antibodies.^5^^,^^6^ Moreover, the global course of the COVID-19 pandemic became less dramatic in terms of disease severity and mortality, not only due to the availability of effective vaccination, but also due to a shift to novel, less aggressive variants of concern (VOCs), predominantly the Omicron (OM) variant and its descendants since early 2022. Because humoral and cellular responses to SARS-CoV-2 vaccination wane over time, booster vaccinations are generally recommended.^7^^,^^8^ We previously found a large proportion of T, particularly those under active anticancer therapy (T_tx_) to launch a “discordant,” i.e., either a humoral or a cellular response, while complete or “concordant” humoral and cellular vaccine failure was rather rare.^5^ We now investigated the humoral and cellular responses to a third mRNA-based booster vaccination in a series of 74 patients with cancer, with focus on specific immunological implications in this vulnerable patient population. Notably, this vaccination series was completed in 2021, i.e., prior to the emergence of the OM wave. This enabled us to study the immunologic anticipation of the new OM variant in patient samples collected prior to this variant’s epidemiologic dominance – and to specifically examine to what extent such major antigenic shift, previously shown to severely compromise antibody (Ab) efficacy,^9^ affected cellular immune responsiveness.

## Results

### Rapid loss of vaccine-mediated protection in patients with cancer

**Figure 1:**
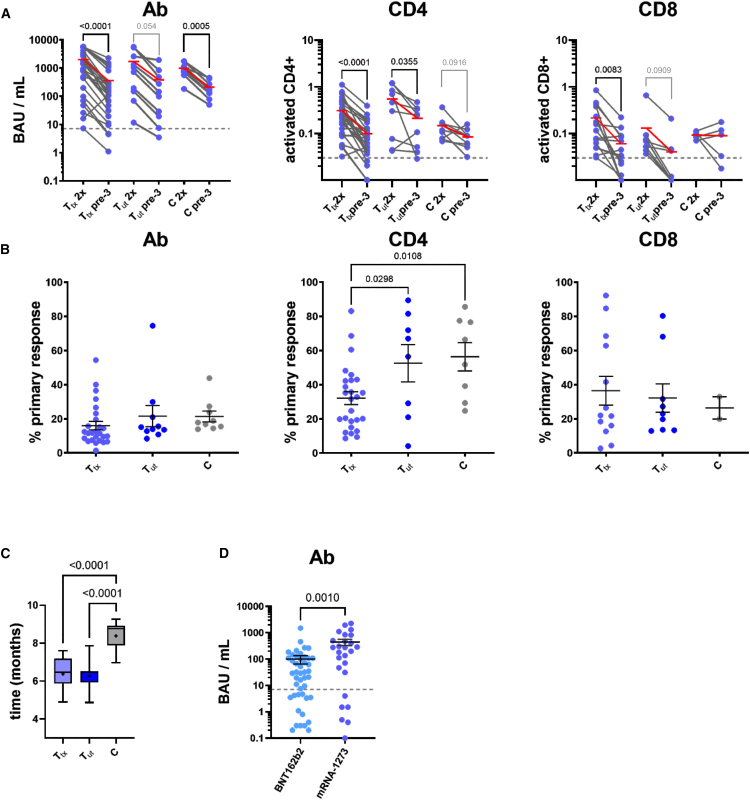
Patients under anticancer therapy exhibit accelerated loss of cellular and humoral vaccination responses (A) Quantitative SARS-CoV-2-specific IgG (left), and spike-activated CD4^+^ (middle) and CD8^+^ (right) responses presented as individual time-course data points (connected by a solid line) between the 2x time-point (after basal double-vaccination) and the pre-boost time-point 5–8 months later (see schematic in Figure S1A for further explanations). Dashed lines indicate thresholds for positive results. Data reflect 27 T_tx_, 10 T_ut_, and 9 C participants. Mean values are highlighted in red. Paired measurements (2x vs. pre-3 time point for each subgroup) were compared using the paired Student’s T-test. *p* values are given in black where statistical significance was obtained (*p* < 0.05), and *p* values between 0.05 and 0.1 are printed in gray. (B) Response waning expressed as comparisons of pre-boost responses normalized to the individual 2x values in the indicated sub-cohorts for the humoral and cellular response types as in A. Error bars indicate mean ± standard error of the mean (SEM). T_tx_, T_ut,_ and C groups were analyzed using one-way ANOVA, and post-hoc comparisons between the subgroups were only performed when significant differences between all groups (*p* < 0.05) were indicated by ANOVA. Numbers above the braces indicate the *p* values of the given comparison (with *p* < 0.05 considered significant). (C) Individual latencies between the second and the third vaccination in the indicated subgroups. Boxplots compare the time span between second vaccination and the booster vaccination (the pre-3 samples were collected immediately before the booster vaccination) for T_tx_, T_ut,_ and C subgroups. Median (line) and mean (cross) are indicated, whiskers indicate data range. Numbers above the braces indicate the *p* values of the given comparison (ANOVA post-hoc comparisons, with *p* < 0.05 considered significant). (D) Pre-boost IgG analyses of 45 participants with cancer that received a BNT162b2-based (left) and 26 participants that received an mRNA-1273-based basal double-vaccination. Unpaired two-sided Student's T-test was used to compare the groups. Error bars indicate mean ± SEM.

Overall, 86 participants (including 12 non-cancer control subjects) were monitored in our study (Table 1). We first compared, in a cohort of 61 participants which had already been included in our previous publication and continued the study for the booster vaccination, humoral IgG Ab as well as cellular CD4^+^ and CD8^+^ T cell responses 3–4 weeks after the second vaccination (“2x”) to the anti-S status just prior (“pre-3”) to the third vaccination 5–8 months later. For these T_tx_ as well as T patients with no concurrent therapy (T_ut_), and 9 control participants (C) with no evidence of cancer (for methodological details see,^5^ and a schematic in Figure S1A), the “2x” Ab, CD4^+^ and CD8^+^ responses from our previous publication served as benchmark for response waning, and are included in Figure 1A (“2x” non-responders were excluded from the analysis; details on results and statistical parameters are provided in Table S1). Across all cohorts, longitudinal comparisons unveiled a decline typically in the range of one to two orders of magnitude, but rarely below the thresholds considered positive for Ab and CD4^+^ responses (Figure 1A, hatched lines). The limited number of cases that displayed retained or even increased levels was most likely explained by exposure to the virus and mild or asymptomatic SARS-CoV-2 infections during this time window, although no consistent pattern across intra-individual Ab, CD4^+,^ and CD8^+^ values was found in these participants. When normalized to the 2x responses (Figure 1B), waning was significantly more pronounced in the T_tx_ compared to the T_ut_ and C group regarding CD4^+^ but not CD8^+^ responses, although the time between the second and the third vaccination was expectedly longer in the non-cancer group, which seemed to have less need for an earlier boost (Figure 1C). Likewise, loss of Ab titers was strongest in the T_tx_ group, but differences were not statistically significant. Of note, CD4^+^ responses declined more slowly than Ab responses, with a mean of 32.1% of the primary CD4^+^ response retained compared to 16.0% for the Ab titer, while CD8^+^ responses presented with high inter-patient variability (Figure 1B; Table S1 for details). Interestingly, when calculated on a per-month basis for each patient (Figure S1B), a reduced decline was observed in the C group compared to the T_tx_ and T_ut_ groups for the Ab response and also between C and T_tx_ groups for the CD4^+^ response. In part, these effects might be due to the earlier booster vaccination in patients and the non-linear waning of Ab and T cell responses. Nevertheless, despite the earlier sampling time-point, retained Ab and CD4^+^ responses were lower in patients with T_tx_ compared to the control group (see mean values marked in red, Figure 1A), indicating quicker waning of responses. Hence, patients with cancer under active antineoplastic treatment presented with a more rapid loss of vaccine-mediated protection, especially evident at the level of CD4^+^ responses. Of note, the pre-boost IgG levels were found to be higher across all participants of the mRNA-1273 vs. the BNT162b2 cohort (Figure 1D), whereas cellular responses exhibited no such difference (Figure S1C).

**Table 1:** Characteristics of the participants

	Patients with Cancer (T)	Control (C)
No. of participants	74	12
Participiants enrolled in previous study^a^	52 (70)	9 (75)
Group characteristics		
Age	–	–
Median (range)	70 (34–84)	45 (31–66)
Body mass index	–	–
Median (range)	25 (17–39)	n.a.
Sex - no. (%)	–	–
Male	31	5
Female	43	7
SARS-CoV-2 and vaccination status - no. (%)		
Seropositive before 3rd vaccination	53 (72)	12 (100)
3rd vaccine dose received	74 (100)	12 (100)
4th vaccine dose received	16	2
Treatment status at time of vaccination no. (%)		
No active treatment (T_ut_)	15	n.a.
Active treatment (T_tx_)	59	n.a.
Tumor type - no. (%)		
Hematologic malignancies	42	n.a.
Lymphoma	22	n.a.
Myeloproliferative neoplasia	9	n.a.
Multiple myeloma	8	n.a.
Other	3	n.a.
Solid tumors	32	n.a.
Breast cancer	8	n.a.
Ovarian cancer	7	n.a.
Gastrointestinal cancer	12	n.a.
Other	5	n.a.
Tumor status - no. (%)		
Complete remission	13	n.a.
Partial remission	14	n.a.
Stable disease	29	n.a.
Progressive disease	16	n.a.
Active disease, not yet response-evaluated	3	n.a.
Stage (solid tumors) - no. (%)		
Localized disease	12	n.a.
Metastasis	20	n.a.
Treatment approach (Solid tumors) - no. (%)		
Adjuvant	5	n.a.
Neoadjuvant	3	n.a.
Palliative	21	n.a.
No active treatment	3	n.a.
Treatment type - no. (%)^b^		
Chemotherapy	23	n.a.
Hydroxyurea	5	n.a.
Therapeutic antibody (not CD20)	10	n.a.
Anti-CD20 antibody	8	n.a.
Small molecule inhibitors	29	n.a.
Endocrine therapy	6	n.a.
High-dose chemotherapy plus auto stem cell transplant	3	n.a.
Companion medication - no. (%)		
High-dose glucocorticoids^c^	5	n.a.
G-CSF	10	n.a.
Vaccination adverse effects - 3rd dose		
None^d^	52	6
Mild	20	4
Moderate	2	2
Severe	0	0
Flu-like/fever	13	6
Fatigue/headache	5	0

a The ‘2x′ data of these patients were published as part of a larger collective in Mairhofer et al., Cancer Cell. 2021 Sep 13;39(9):1171–1172. https://doi.org/10.1016/j.ccell.2021.08.001.

b Added percentages of diverse treatment types may exceed 100%, since some patients received combination therapies.

c High-dose glucocorticoids: prednisolone equivalent >10 mg/day.

d Weak pain and short-lived local irritations at site of injection were not considered as adverse effects.

### Booster vaccination profoundly enhanced anti-S Ab and CD4^+^ T cell responses

**Figure 2:**
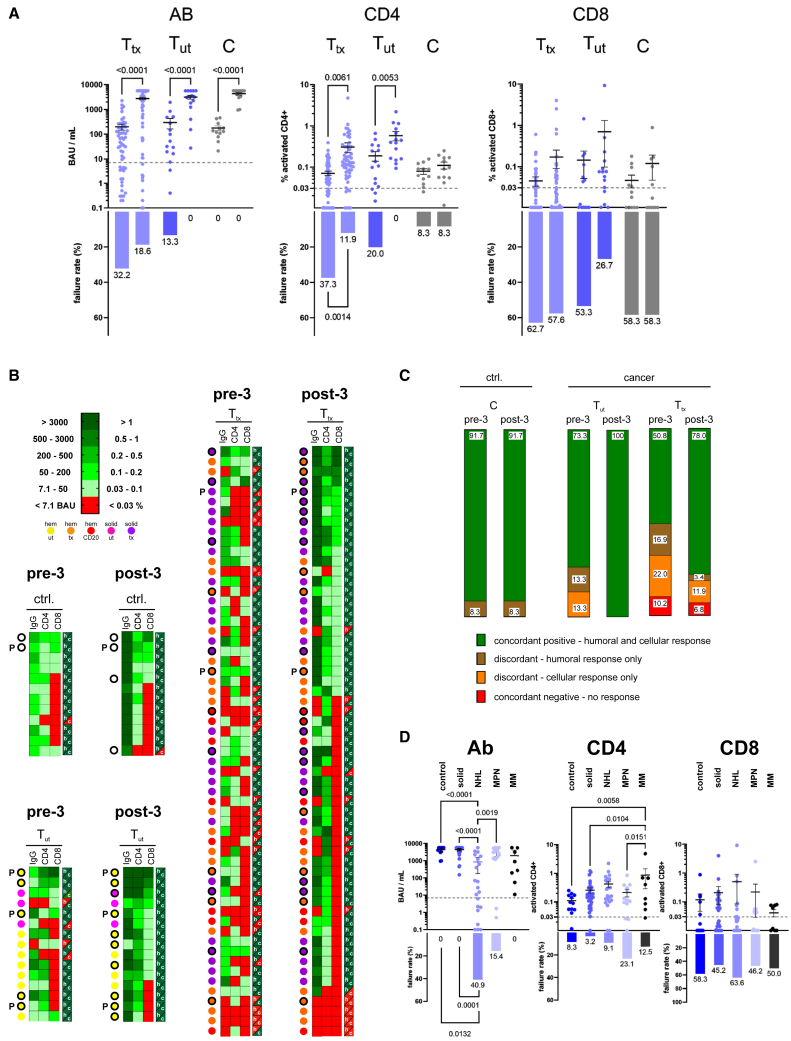
Efficacy of a booster vaccination in patients with cancer (A) Quantitative SARS-CoV-2-specific IgG, and spike-activated CD4^+^ and CD8^+^ responses in the T_tx_ (light-blue, left), T_ut_ (dark-blue, middle), and C (gray, right) sub-groups, comparing pre-boost (left) to post-boost (right) response data (top). Dashed lines indicate thresholds for positive results; thus, values below are considered negative and contribute to the percentages of failures in the indicated response categories (bottom). Data reflect 59 T_tx_, 15 T_ut,_ and 12 C participants. Error bars indicate mean ± SEM, and numbers above the braces indicate the *p* values of the given comparison (paired Student’s T-test, with *p* < 0.05 considered significant). Failure rates are depicted below and were compared using Fisher’s exact test. Significant differences (*p* < 0.05) are indicated on the graph. (B) Heatmaps summarizing humoral and cellular responses measured before (pre-3) and after (post-3) the booster vaccination. Different patient with cancer groups are color-coded to represent the quantification and tumor type/treatment designation. On the right of each column, green and red indicate positive and negative humoral (h) and cellular (c) responses, respectively. Concordant results are h/c green or red; discordant results are color-mixed. Confirmed PCR positivity is indicated by “P” beside the color code, and M/N positivity (see Figure 3) is marked by a black circle. The indicated groups consist of 12 C participants, and 15 T_ut,_ and 59 patients with T_tx_ with cancer. To facilitate comparisons, participants in the pre-3 and post-3 heatmaps are shown at identical positions/order. (C) Fractions of participants with concordant or discordant humoral (IgG) and cellular (either CD4^+^ or CD8^+^ or both) responses. The percentages are based on individual data triads as shown in (B). (D) Antibody and cellular (CD4^+^ and CD8^+^) responses from Figure 2A split into controls and different patient subgroups (solid – all solid tumors, NHL – non-Hodgkin lymphomas, MPN – myeloproliferative neoplasms, MM – multiple myeloma). Differences between subgroups were analyzed using one-way ANOVA. Numbers above the braces indicate the *p* values of the given comparison (with *p* < 0.05 considered significant). Failure rates are depicted below, and were compared as outlined in (A). Of note, compared to Figure 1, which focuses on waning responses, Figure 2 is dedicated to “booster efficacy,” which implies that some data are contributed to both panels.

Next, we analyzed the post-boost results. Of our 86 participants, i.e., an expanded cohort of 59 T_tx_, 15 T_ut,_ and 12 C, 82 received a booster vaccination with mRNA-1273 at an initial dose level (reflecting the basal immunization dose of 100 μg, which was later amended to a lower dose of 50 μg for booster application^10^; two participants from the C group already received the lower dose, and two received a BNT162b2 booster due to the temporal non-availability of mRNA-1273. Humoral and cellular responses were analyzed 3–4 weeks after the booster vaccination (Figure S1A). When comparing pre-to post-boost responses, patients with cancer presented with a significant increase of their spike-specific IgG levels (13.9-fold on average for T_tx_ and 10.6-fold for T_ut_) and CD4^+^ T cell frequencies (4.3--fold on average for T_tx_ and 3.1-fold for T_ut_), with a similar trend but a large proportion of non-responders observed regarding the spike-specific CD8^+^ T cell reactivity (Figure 2A; analysis results and statistical parameters are provided in Table S2). Accordingly, CD8^+^ T cell responses, which scored negative in 58% of the patients prior to the third vaccination, remained below threshold for more than half of the patients after the booster. In contrast, the fractions of patients with T_tx_ with formally negative IgG or CD4^+^ responses just prior to the boost (failure rate [FR] of 32.2% and 37.3%, respectively) became much smaller after the third vaccination (FR of 18.6% and 11.9%, respectively). Notably, absolute levels reached upon the boost by patients with cancer were similar to, or, with respect to cellular responses, even higher than those of control participants.

When plotted as individual heatmaps color-coded for humoral and cellular response levels across the T_tx_, T_ut_, and C cohorts prior to and after the booster vaccination, and further informed by the tumor type (solid vs. hematological), we observed non-responders *(*i.e., no humoral and no CD4^+^ or CD8^+^ T cell response) in none of the C or T_ut_ participants, but in a small subset of patients with T_tx_. These non-responders presented almost exclusively with hematologic malignancies (5/6 pre-boost and 4/4 post-boost, Figure 2B. Note that all participants are shown as matched pre-3/post-3 datasets at the same position in the corresponding heatmaps). While two of these four patients also presented with below-threshold humoral and cellular levels pre-boost (Figure 2B, see T_tx_ pre-3), four out of six patients with negative pre-boost humoral and cellular reactivity achieved at least a humoral or a cellular response to the booster vaccination (Figure 2B, see T_tx_ post-3). Notably, as we reported for patients with cancer after basal SARS-CoV-2 vaccination,^5^ we also found a large fraction of discordant responders (i.e., only a humoral or a cellular T cell response) in the T_tx_ and T_ut_ groups prior to booster vaccination. This proportion was strongly reduced in T_tx_ after the booster dose, no longer detectable in T_ut,_ and remained low in the C group (Figure 2C). All T_ut_, and more than three-quarters of the T_tx_ participants achieved concordant responses to the booster vaccination. Thus, most patients with tumor, even under active anticancer treatment, exhibited improved responsiveness to the booster vaccination.

When comparing different tumor types, no obvious differences were observed between patients with solid tumors and the C group (Figure 2D; see Table S2 for details). However, patients with Non-Hodgkin’s lymphomas (NHL) exhibited a significant FR of 40.9% (i.e., 9 out of 23) regarding an IgG response, while control and solid tumor groups presented failure-free. Notably, all patients with multiple myeloma (MM) and all except two with myeloproliferative neoplasms (MPN) produced a humoral response. Collectively, these data indicate a very high likelihood for C and T_ut_ participants to lift their above-threshold pre-boost response levels further by a third vaccination. Patients with T_tx_, especially with hematologic malignancies, and here CD20 Ab-pretreated NHL in particular, failed more frequently to launch an Ab response, but typically achieved a robust CD4^+^-based T cell response.

### SARS-CoV-2 infections enhance vaccine-evoked protection

**Figure 3:**
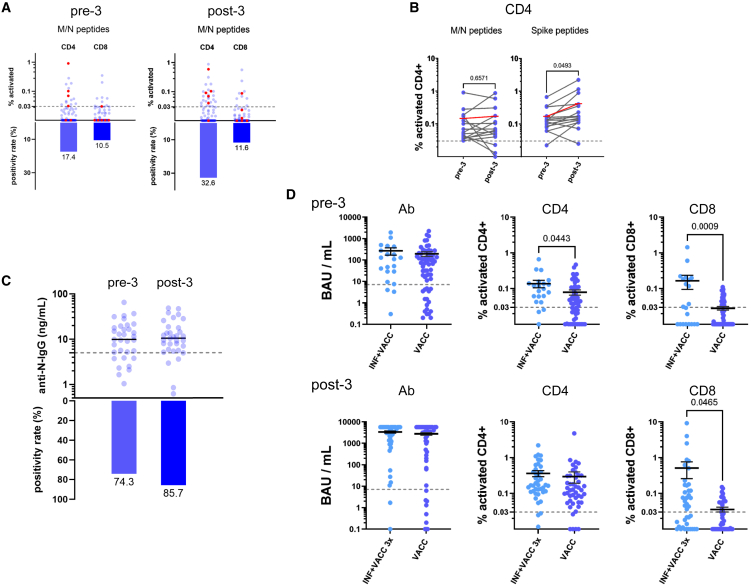
M/N reactivity in T cell assays after the booster vaccination indicates virus contact/asymptomatic infections with augmented humoral and cellular responses (A) Results for M/N peptide stimulations indicating virus exposure and/or asymptomatic infections (T_tx_, T_ut,_ and C aggregated in one plot for CD4^+^ and CD8^+^ T-cells, *n* = 85) prior to (pre-3) and after booster vaccination (post-3). Positivity rates for CD4^+^ and CD8 are indicated below. Participants with PCR-confirmed virus contact (prior to the beginning of the vaccination campaign) are highlighted in red. (B) Comparison of M/N and spike peptide CD4^+^ T cell activation before (pre-3) and after booster vaccination (post-3) in all patients who showed M/N reactivity prior to the booster vaccination (*n* = 15). M/N reactivity is not significantly altered, whereas spike reactivity significantly increases (as expected by spike mRNA immunization). Numbers above the braces indicate the *p* values of the given comparison (paired Student’s T-test, with *p* < 0.05 considered significant). Mean values are indicated in red. (C) Humoral anti-nucleocapsid (N) IgG ELISA measurements confirm prior virus contacts. Paired plasma samples of 33 participants demonstrating cellular M/N reactivity at least at one time point, all stored at −80°C since collection either prior to (pre-3) or approximately 3–4 weeks after the booster vaccination (post-3), were thawed, and N antibodies were determined using the Legend-Max-SARS-Cov-2-Nucleocapsid-human-IgG ELISA kit as outlined in the STAR Methods. The threshold for humoral N positivity was set to 5 ng/mL as marked by the dotted line. The bars below indicate the fractions of N-positive samples. (D) Comparison of virus-exposed and vaccinated individuals (INF+VACC), as indicated by PCR-confirmed infection or M/N positivity before the booster vaccination (pre-3, *n* = 21) to all others without evidence for virus contact (VACC) reveals significantly higher CD4^+^ and CD8^+^ T cell responses. After the booster vaccination (post-3, *n* = 40 INF+VACC), only CD8^+^ T cell responses are significantly increased compared to INF+VACC to VACC. Unpaired two-sided Student's T-test was used to compare the groups. Error bars indicate mean ± SEM.

T cell responses against the viral M (membrane) or N (nucleocapsid) proteins, which are not encoded by the spike-restricted mRNA vaccines, are a strong indicator of a preceding exposure to SARS-CoV-2, and are not induced by infections with other endemic coronaviruses such as OC43, HKU1, NL63 or 229E.^11^^,^^12^^,^^13^ We wondered whether M/N reactivity might correlate with reduced waning at the pre-boost time-point. We included SARS-CoV-2 M/N peptide stimulations in our pre- and post-boost measurements to compare positive CD4^+^ or CD8^+^ cellular responses to these non-vaccine-encoded viral peptides across participants (Figure 3A). Despite the short interval of no more than one month between the collection of the samples, the proportion of M/N-reactive and/or PCR-confirmed samples almost doubled similarly across all subgroups (C, T_ut_, T_tx_) from 24.4 to 46.5% (Table 2). This most likely reflects 19 unrecognized SARS-CoV-2 infections within these 3–4 weeks, 17 of them occurring in the T groups. Such numbers are remarkable when considering all the non-pharmaceutical interventions in force in 2021/22, and PCR- and antigen-based test assays were widely available free-of-charge. When analyzing the C, T_ut_, and T_tx_ subgroups, T_ut_ displayed the highest fraction of M/N reactivity (66.7%), with T_tx_ (44.1%) and C (33.3%) scoring considerably lower. This could indicate a potential confounding effect by unnoticed infections that may have evoked particularly strong cellular immune responses in the T_ut_ group, thereby contributing to the lower failure rate observed for CD4^+^ and CD8^+^ responses (Figure 2B; pre-3 and post-3 heatmaps mark M/N reactivity as black circles and PCR-confirmed infections as “P” beside the tumor type-encoding colored dot).

**Table 2:** M/N positivity and PCR confirmed infections

		Positive	Negative	% Positive	n	p (Fisher's exact test)
T_tx_	pre-3	14	45	23.7	59	0.032
	post-3	26	33	44.1		
T_ut_	pre-3	5	10	33.3	15	0.143
	post--3	10	5	66.7		
C	pre-3	2	10	16.7	12	0.640
	post-3	4	8	33.3		
all	pre-3	21	65	24.4	86	0.004
	post-3	40	46	46.5		

When pre-3 peripheral blood mononuclear cells (PBMC) were stimulated with pooled M and N peptides, above-threshold CD4^+^ or CD8^+^ T cell activation was detected across all groups in 17.4% and 10.5% of the participants, respectively (Figure 3A; six participants with PCR-confirmed previous infections are highlighted in red). Next, we compared the effects of the booster vaccination in the samples that presented with M/N reactivity already prior to the booster. As expected, the spike-based mRNA vaccine had no significant effect on CD4^+^ M/N reactivity, whereas CD4^+^ S reactivity was significantly increased (Figure 3B). To confirm the cellular immune memory of a potential SARS-CoV-,2 exposure at the humoral level, cryopreserved plasma samples from patients with M/N-reactive (*n* = 33) were analyzed for the presence of anti- N IgG antibodies using an ELISA kit (Figure 3C). N IgG antibodies were detected in 74.3% of the pre-3 samples, and increased to 85.7% in the post-3 samples. Incomplete overlap between cellular M/N and humoral N reactivity was potentially due to broader, S- and N-affecting humoral non-responsiveness in the respective groups, but first and foremost explained by a much higher rate of formally positive N-reactive IgG results, raising concerns about the validity of this assay with its undetermined specificity and sensitivity, and its potential cross-reactivity with Ab against non-SARS-CoV-2-originated N proteins from seasonal human coronaviruses.^14^ When comparing the spike-specific Ab, CD4^+^ and CD8^+^ responses of participants with PCR-confirmed infections or positive M/N reactivity (termed “INF+VACC,” known as “hybrid immunity”^15^) prior to or after the boost (termed “INF+VACC 3x”) to other participants, a significant increase was observed in both T cell subsets from hybrid-immune participants prior to the boost (Figure 3C; INF+VACC vs. VACC in pre-3). Notably, activated CD8^+^ T-cells were also found significantly higher in those individuals after the third vaccination (Figure 3C; INF+VACC or INF+VACC 3x, vs. VACC, respectively, in pre-3 and post-3), whereas no differences in their spike-specific IgG levels were observed (see Table S3 for details). Hence, actual virus encounter boosted cell-based anti-spike immunity in patients with cancer. This further underscores an increased cellular immune response induced by a combination of vaccination and virus contact, even if the virus impact was asymptomatic. Notably, virus-evoked T cell responses may be even broader, and, besides M/N peptide reactivity, might include reactivity to non-structural virus proteins not assayed here.^16^

### Wild-type spike-evoked cellular immunity remains reactive to omicron-specific mutations

**Figure 4:**
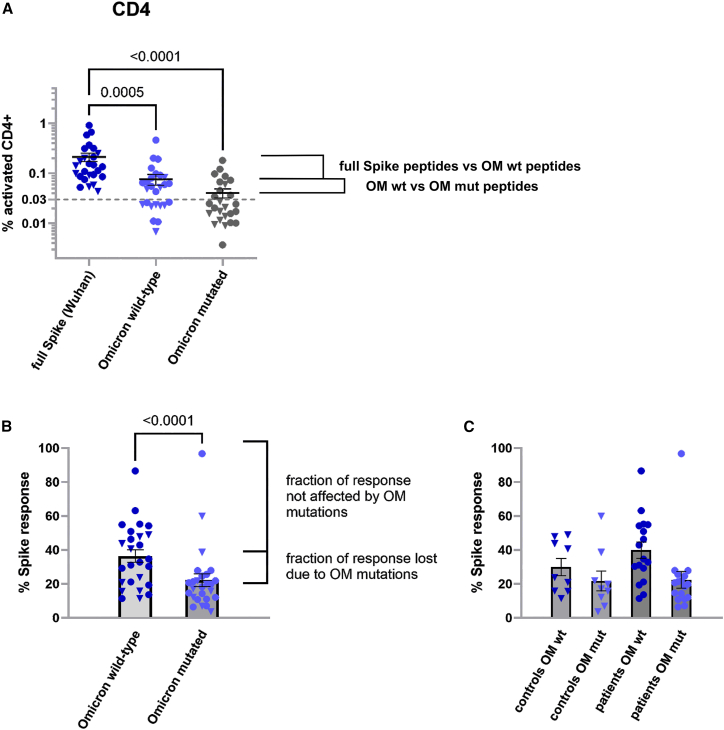
Anticipation of Omicron (OM) reactivity in booster vaccinated patients prior to the OM wave (A) PBMCs of selected participants (*n* = 26) with a strong cellular immune response prior to the booster vaccination were stimulated either with the full spike peptide complement (left), or with only the peptides potentially mutated in the OM variant, either with their wild-type (middle) or mutated sequences (right). Activated CD4^+^ T-cells are graphed. Numbers above the braces indicate the *p* values of the given comparison (ANOVA post-hoc comparisons, with *p* < 0.05 considered significant). Control samples are indicated by triangles throughout the figure. (B) The OM wild-type and mutated measurements are normalized to the full spike peptide responses, visualizing which fraction of the response is lost either due to the reduced peptide complement or specifically due to the numerous OM mutations. Paired Student's T-test was used to compare the groups. (C) The normalized measurements were further split into controls (*n* = 9) and patients (*n* = 17) to reveal potential differences. Error bars indicate mean ± SEM in all panels.

Spike protein-affecting mutations in emerging variants of concern (VOC) may allow escape from vaccination-mediated virus control. Hence, we asked whether and how booster vaccination might affect cell-based immunity toward the massively emerging VOC Omicron (OM) B.1.1.529 even prior to its actual dominance in Austria in early 2022, i.e., before individual encounters with OM-typical spike protein mutations could occur.^17^ To investigate OM-specific immune responses shortly after the third vaccination (“post-3”; obtained prior to the OM wave), we analyzed a subset of 22 selected participants who had exhibited particularly strong spike protein-directed CD4^+^ responses in their pre-booster samples. Of these participants, post-3 PBMC samples were stimulated with peptide pools covering all amino acids mutated in the OM VOC, either as wild-type (wt) or mutant (mut) peptides. The measured levels of T cell activation were compared to the full-length spike-covering peptide stimulations in the same patients, thereby allowing us to determine the extent to which OM mutations individually impaired cell-based spike-reactivity. Both OM wt and OM mut pools elicited significantly reduced CD4^+^ responses compared to full spike, indicating that a sizable fraction of CD4^+^ T-cells was activated by peptides not present in the OM pools, irrespective of OM-specific mutations, and, thus, not affected by OM mutations (Figure 4A; see Table S4 for details). To quantify this effect, OM wt and OM mut responses were normalized to the full spike response and their mean values were compared. Approximately 38% of the full spike reactivity was retained after activation with the OM wt peptides, whereas only 22.5% remained when challenged with OM mut peptides (Figure 4B). A similar association of CD4^+^ T cell responses with peptides fully conserved in OM was previously reported for a small number of healthy probands after two vaccine doses and for unvaccinated convalescent probands.^18^^,^^19^ Of note, we found a relatively high variance among the participants, with some individuals showing much lower or higher retention of reactivity, indicating clonal differences in the individual T cell responses, but with no significant differences in spike-normalized responses between patients with cancer and controls (Figure 4C). CD8^+^ responses followed the same trend, albeit with even higher inter-sample variability (Figure S2). Collectively, these analyses highlight an important aspect of the initial Wuhan-Hu-1 strain spike-protein-based mRNA vaccination regarding the T cell activation by many peptides outside mutated regions as found in OM, but also a preserved but significantly reduced reactivity to peptides now altered by VOC-specific mutations. Our measurements demonstrate that more than 50% of the CD4^+^ and CD8^+^ reactivity detected *in vitro* refers to peptides that are not affected by OM mutations. Therefore, escape from cellular immunity is not as easily achieved as compared to humoral immunity, where OM mutations reportedly impaired Ab-mediated virus neutralization.^20^^,^^21^ Notably, after three vaccinations, the majority of our vulnerable patients with cancer mounted a spectrum of cell-based responses directed against various regions of the Spike protein, albeit with some restraints depending on disease type and treatment status.

### Augmented seroconversion rates in repeatedly vaccinated patients with cancer

**Figure 5:**
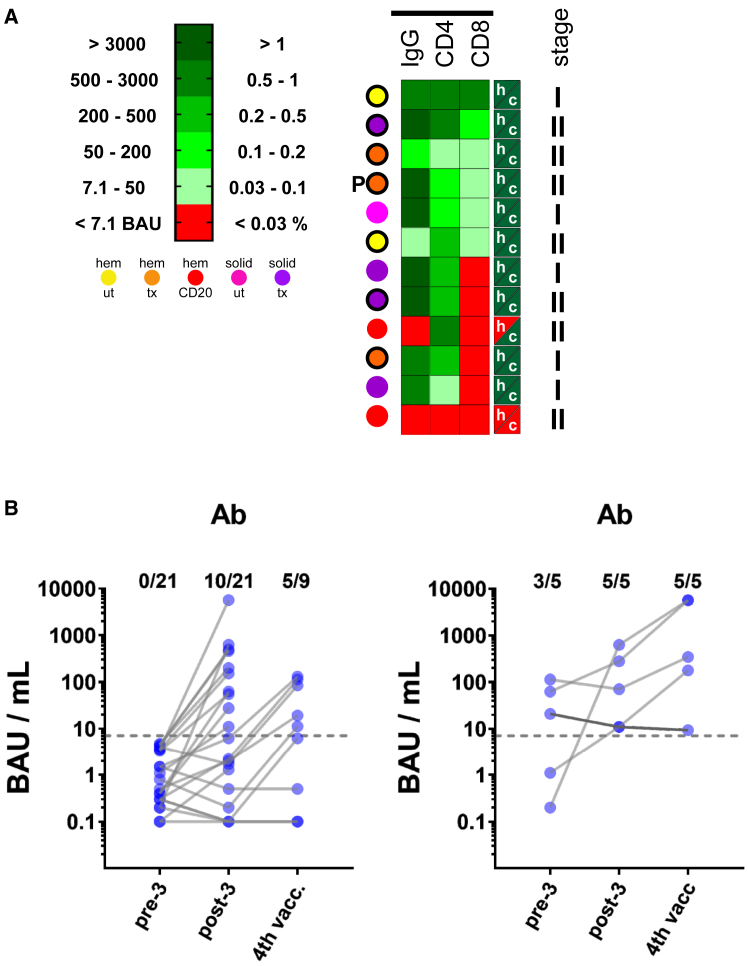
Confirmed breakthrough infections within our patient collective and fourth vaccination in seronegative/low-titer patients (A) Post-booster heatmap for the 12 confirmed COVID-19 cases until the end of April 2022. (B) Left panel: Antibody titers of seronegative patients who either seroconverted after the booster vaccination or after they received a fourth vaccine dose 1–2 months after the boost dose. Right panel: Effect of a fourth vaccine dose on Ab titers in patients who already displayed low positive titers pre or post-boost.

In a follow-up period of 2–3 months after the booster vaccination, 12 rapid antigen test- or PCR-confirmed SARS-CoV-2 breakthrough infections were registered among the participating patients with cancer. According to a 2020 consensus on COVID-19 disease severity,^22^ all 12 test-positive cases were categorized as low-stage infections, encompassing stage I (i.e., subclinical or asymptomatic infections only recognized by antigen or PCR testing; *n* = 5) and stage II (i.e., mild to moderate symptoms; *n* = 7; Figure 5). When analyzing the Ab and T cell responses (extracted and regrouped from Figure 2A for better visualization), virtually all were Ab seropositive (i.e., 10 out of 12), and showed CD4^+^ T cell reactivity (i.e., 11 out of 12). Of note, one patient who received anti-CD20 Ab treatment in the context of a hematologic malignancy did not seroconvert and showed no T cell reactivity, but overcame the viral infection, presumably based on the OM VOC, with only mild symptoms. Interestingly, only 2 out of 5 stage I infections occurred with documented previous virus exposure (bold circles in Figure 5A), whereas 5 out of 7 stage II infections presented with or despite an M/N-positive history. All patients with stage I infection had a robust Ab response (>500 BAU/mL) and a positive CD4^+^ response (Figure 5A).

Patients with no or low Ab responses after the booster vaccination were offered a fourth dose of mRNA-1273 four to eight weeks later. Prior to the booster vaccination, 21 patients had an insufficient Ab response. Of these patients, ten seroconverted after the third vaccination, and of the nine (out of eleven) seronegative patients who accepted the fourth dose, five seroconverted thereafter (Figure 5B, left panel). Five patients who had seroconverted but showed titers below 500 BAU/mL also received a fourth dose (Figure 5B, right panel). Responses varied, but high titers were finally detected for two of these patients. These results indicate that seroconversion can be achieved with repeated booster shots in a large proportion of patients with cancer despite an initial lack of seroconversion.

## Discussion

Our investigation of humoral and cellular immune responses to an mRNA-based third or booster SARS-CoV-2 vaccination is one of the largest, longitudinal studies among the particularly vulnerable population of patients with cancer. Following-up on our initial report on basal SARS-CoV-2 immunization in patients with cancer,^5^ we found cellular responses to be more lasting than the more rapidly waning Ab responses. Importantly, the booster vaccination effectively countered the loss of immune reactivity over time, yielded responsiveness in patients that failed before and significantly lowered the fraction of patients with cancer under antineoplastic treatment with “discordant” (i.e., only humoral or cellular) responses to the initial vaccination from the previously reported 22.6% (mRNA-1273 group only for proper comparison) to now 15.3%, increasing the proportion of concordant positive patients from 67.7% to 78%. Including also patients with cancer in need of a fourth vaccination due to insufficient responsiveness after preceding vaccinations, our study underscores the general ability of patients with cancer, even under active antineoplastic therapy, to mount an immune response upon vaccination. Unsuccessful attempts may be overcome by additional vaccine doses, eventually leaving only a small proportion of patients with cancer with “vaccine failures,” i.e., with a concordant lack of both a humoral and cellular anti-spike response.

Our study is enhanced by additional information on previous exposure to SARS-CoV-2, which we obtained by measuring cellular reactivity against structural viral proteins – the M and N proteins – that cannot be elicited by the spike-protein-restricted mRNA vaccine. Quantifying lastingly M/N-reactive T-cells is a particularly meaningful strategy to detect asymptomatic infections that were not otherwise registered by only temporarily positive antigen- or PCR-based assays. Our results indicate that mRNA-based vaccination is more effective, especially regarding a CD8^+^ T cell response, when administered to individuals with actual virus experience, a phenomenon known as hybrid immunity in general and here confirmed for patients with cancer in particular. Patients with vaccination-plus-infection-primed hybrid immunity also harbor T-cells that can recognize presented peptides from M, N, and other viral proteins in addition to the spike-confined immune response to the vaccination alone, further rendering immune escape more unlikely.^13^^,^^15^^,^^23^^,^^24^ Our study was not designed to unveil the precise mechanism by which infection and vaccination interfere with each other, since the exact moment when the infection occurred remained uncertain in most participants. Preceding infections may prime for a stronger vaccination response, while post-vaccination virus encounter might attenuate its decline. Unexpectedly, M/N reactivity almost doubled between the third vaccination and the next sample collection just a few weeks later. Notably, an ELISA-based survey of Ab-based N reactivity in these patients revealed an increased humoral response matching our finding regarding a cellular M/N memory, but the much higher rate of participants with N-reactive Ab raised concerns about this assay’s SARS-CoV-2 specificity.^14^ The increased cellular M/N reactivity shortly after three vaccinations indicates the real-world incapability of non-pharmaceutical interventions and probably booster vaccinations as well of preventing virus spread. Underscored by the typically mild or even clinically inapparent infections that occurred unrecognized in our mRNA-vaccinated collective of vulnerable patients, this reflects an insufficiency of the IgG-based Ab against S and N proteins to provide protective mucosal immunity against virus entry at the upper respiratory tract,^25^ and a stronger reliance on T cell immunity due to the intracellular protein processing and cell surface presentation of immunogenic peptides within major histocompatibility complexes, upon which intracellular virus replication competes with T-cell-based cytolysis.^26^^,^^27^

With the exception of five patients, all booster vaccinations were completed prior to reporting of the first Omicron case in Austria on November 28, 2021. All post-3 samples for OM responsiveness were collected before the national lockdown because of the Delta VOC-wave was relieved in mid-December, thereby rendering any actual OM interference highly unlikely. This timing allowed us to conduct conceptually interesting analyses on “anticipatory” responsiveness toward the OM-specific mutant spike protein in individuals vaccinated with a Wuhan-Hu-1 spike-encoding mRNA. Using peptide pools specific for OM spike protein mutations, we found the majority of detectable cell-based anti-spike reactivity to recognize peptides outside the respective OM-affected sites. To our surprise, we detected only slightly reduced T cell reactivity against an OM-restricted pool of mutated as compared to wt spike peptides, thereby challenging the view of OM mutations as cellular immune escape variants. Conversely, our results underscore the importance of a robust cellular immunization against the wt spike protein, as T-cells will still effectively recognize and counter future emerging VOC by destroying virus infected cells via presentation of immunogenic viral peptides on their surface. The functional value of such anticipatory responsiveness asks for confirmation in epidemiological studies including vulnerable groups such as patients with cancer in light of the currently circulating OM-derived strains, now considered “variants of interest” or “VOC under surveillance” by the WHO. Of note, we specifically investigated SARS-CoV-2 immune responses in patients with cancer with a focus on the vaccination strategy in retrospect. Nevertheless, our findings may have important implications for future endemic or pandemic virus challenges with other highly mutagenic viruses, especially to better protect patients with cancer via guided vaccine adaptations.^28^

### Limitations of the study

The main limitations of our study are the relatively small sample sizes when comparing/analyzing healthy controls and different types of cancer as well as the limited sensitivity of detection of activated CD8^+^ T-cells in peripheral blood samples.

## Resource availability

### Lead contact

Requests for further information and resources should be directed to and will be fulfilled by the lead contact, Clemens A. Schmitt (clemens.schmitt@charite.de; clemens.schmitt@kepleruniklinikum.at).

### Materials availability

This study did not generate new unique reagents.

### Data and code availability

• All data reported in this article will be shared by the lead contact upon request.
• This article does not report original code.
• This article did not create any other items which are available for sharing with the scientific community.

## Acknowledgments

This study was supported by the Förderverein Hämatologie und internistische Onkologie (Tyle Private Foundation, Linz, Austria), and the Clinician Scientist Program of the JKU to L.K. and M.S. All patients and volunteers gave their informed consent to participate in the analyses. We express our sincere gratitude for their active involvement.

## Author contributions

M.M. T cell activation experiments, data analysis, preparation of figures and tables, writing of article; L.K. patient recruitment, sample collection and follow-up, data curation, contributed to the writing and revision of article; S.K. sample processing and revision of article; M.P. sample processing; R.G. administration and IRB submission; M.S. sample processing and revision of article; C.A.S. Conceptualization, supervision, funding acquisition, and writing of article.

## Declaration of interests

C.A.S. received travel support, honoraria, and consulting fees from Abbvie, AstraZeneca, Bayer, Beigene/BeOne, Bristol-Myers-Squibb/Celgene, Eli Lilly, Gilead/Kite, Janssen-Cilag, MSD, Novartis, Octapharma, Pfizer, Pierre Fabre, Roche, Sanofi, and Takeda.

## STAR★Methods

### Key resources table

**Table:** 

REAGENT or RESOURCE	SOURCE	IDENTIFIER
**Biological samples**		

Human PBMCs & plasma samples	Blood drawn from tumor patients and healthy controls	IRB 1070/2020

**Chemicals, peptides, and recombinant proteins**		

PepTivator® SARS-CoV-2 Prot_S	Miltenyi Biotech	Cat#130-127-041
PepTivator® SARS-CoV-2 Prot_S1	Miltenyi Biotech	Cat#130-126-701
PepTivator® SARS-CoV-2 Prot_S+	Miltenyi Biotech	Cat#130-127-311
PepTivator® SARS-CoV-2 Prot_M	Miltenyi Biotech	Cat#130-126-703
PepTivator® SARS-CoV-2 Prot_N	Miltenyi Biotech	Cat#130-126-698
PepTivator® SARS-CoV-2 Prot_S B.1.1.529/BA.1 Mutation Pool	Miltenyi Biotech	Cat#130-129-928
PepTivator® SARS-CoV-2 Prot_S B.1.1.529/BA.1 WT Reference Pool	Miltenyi Biotech	Cat#130-129-927

**Critical commercial assays**		

Antigen-specific T cell Analysis Kit (PBMC), anti-human, REAfinity™	Miltenyi Biotech	Cat#130-138-375
LegendMAX SARS-CoV-2 Nucleocapsid human IgG ELISA kit	BioLegend	Cat#448107

**Software and algorithms**		

Graphpad Prism	GraphPad Software	Version 10.4.2
SPSS	IBM	Version 29.0.0.0(241)
Kaluza	Beckman-Coulter	Cat#A82959
Rstudio	Posit software	2025.05.0 build 496
R		https://www.R-project.org/
R package “drc”	1)	https://CRAN.R-project.org/package=drc
R package “dplyr”		https://doi.org/10.32614/CRAN.package.dplyr

**Other**		

CytoFLEX V5-B5-R3 Flow Cytometer (13 Detectors, 3 Lasers)	Beckman-Coulter	Cat#B53000
SpectraMax ABS Plus Absorbance ELISA plate reader	Molecular Devices	N/A

1) Ritz C, Baty F, Streibig JC, Gerhard D (2015). “Dose-Response Analysis Using R.” _PLOS ONE_, ∗10∗(e0146021). <http://journals.plos.org/plosone/article?id=10.1371/journal.pone.0146021>.

### Experimental model and study participant details

#### Patients with cancer and control participants

Beginning in March 2021 with the first dose and a booster dose three to four weeks later, the Kepler University Medical Center Linz, Austria, was able to offer patients with cancer 18 years of age or older as part of a particularly vulnerable population at risk of more severe COVID-19 courses in a first series BNT162b2-based, in a second series mRNA-1273-based SARS-CoV-2 vaccination. As booster vaccine, mRNA-1273 was offered to the patients 4–6 months after the initial two vaccine doses. This tumor patient cohort (termed group T) included patients with solid and hematologic malignancies (with the exception of non-melanoma skin cancers) without systemic anticancer therapy in the last three months and without anti-CD20 therapy in the last six months (subgroup T_ut_) or with recent or current anticancer therapy (subgroup T_tx_). Vaccination controls (subgroup C) consisted of staff working at the Kepler University Medical Center with no record of solid or hematologic malignancies during the last three years who received two doses of BNT162b2 starting January 2021, and were also offered the mRNA-1273 booster 6–8 months after the second vaccine dose.

#### Blood samples

Sample collection from patients with cancer, non-cancer controls and convalescent COVID-19 patients was approved by the ethics committee/institutional review board of the Medical Faculty of the Johannes Kepler University Linz, with permit number 1070/2020 and its subsequent amendments in 2021 and 2022. For detailed patient characteristics, see Table 1. After obtaining informed consent, venous blood was collected for analysis of serum Ab levels and isolation of peripheral blood mononuclear cells (PBMC) immediately prior to the booster vaccination and 3–4 weeks after the vaccination. PBMC aliquots were stored at −80°C until analysis.

### Method details

#### PBMC isolation

For PBMC isolation, blood was drawn into Vacutainer NH-CPT tubes (BD Biosciences) and centrifuged at 1650x g for 20 min at room temperature. Plasma aliquots were collected for each donor and stored frozen at −80°C. The buffy coat was collected from the top of the gel and diluted 1:1 with PBS. PBMC were pelleted (300x g, 10 min), residual red blood cells were removed by resuspension of the pellet in 5 mL NH_4_Cl lysis buffer and incubated for 6 min at room temperature. Tubes were filled with PBS to 10 mL, centrifuged, washed once in 10 mL PBS, then resuspended in 5 mL PBS. An aliquot was removed for viable cell count, cells were pelleted (300x g, 5 min), resuspended in freezing medium (fetal bovine serum with 10% dimethyl sulfoxide [DMSO]), aliquoted in cryotubes and frozen down by using polystyrene foam containers that provided a constant cooling rate of −1 °C/min. Samples were stored at −80°C until T cell measurements were carried out.

#### Quantification of SARS-CoV-2 spike-specific antibody titers

IgG antibodies recognizing the receptor binding domain (RBD) of the spike protein were quantified using a CE-IVD-certified chemiluminescence-microparticle-based assay (Abbott) according to the manufacturer’s instructions. Chemiluminescence measurements were performed using the proprietary ARCHITECT system (Abbott). Raw measurements were converted into binding antibody units (BAU)/mL according to WHO standards. The cut-off for sample positivity was set by the manufacturer to 7.1 BAU/mL, reflecting a 50% inhibitory dose (ID50) at a dilution of 1:20 in a plaque reduction neutralization test with 95% probability, and the upper detection limit of the assay was 5.680 BAU/mL.

#### Flow cytometric quantification of SARS-CoV-2 spike-specific activated CD4^+^ and CD8^+^ T-cells

Spike-specific activated T-cells were quantified using the SARS-CoV-2 Prot_S human T cell Analysis Kit (Miltenyi Biotech). To maximize spike-specific responses, S1 and S+ Peptivator pools were admixed to the Prot_S Peptivator pool included in the kit. To determine M/N reactivity, PBMCs were analogously stimulated with mixed M and N peptivator pools. Sensitivity of responses to spike mutations in the OM SARS-CoV-2 variant were assayed by stimulation of PBMCs with a pool of mutated peptides compared to the corresponding non-mutated peptides and complete spike peptides (S, S1 and S+ peptivators pooled). Peptides were added to each participant’s individual PBMC population, of which the antigen-presenting cells therein take up the peptides, process and present them via major histocompatibility class I or II molecules to autologous CD4^+^ or CD8^+^ T-cells. Cryopreserved PBMC were thawed and allowed to recover over-night in TexMACS medium (Miltenyi Biotech). Viable PBMC were quantified, and approximately 1 × 10^6^ cells were used for T cell stimulation and staining according to the manufacturer’s instructions. In brief, 100 μL of each PBMC sample were dispensed into 96-well plates, and 6 μL of peptides solubilized in 10% DMSO were added. For each sample, cells supplemented with 6 μL of 10% DMSO in milliQ water were included as a negative control. After 2 h of stimulation at 37°C and 5% CO_2_, 2 μL of Brefeldin A (100 μg/mL) to inhibit intracellular protein transport were added, and incubation was continued for another 4 h. Then, cells were incubated with a viability dye for 30 min, fixed, permeabilized and stained with the eight antibodies contained in the kit. After a final washing step, cell pellets were resuspended in 200 μL of PEB buffer (i.e., PBS, pH 7.4, supplemented with 2 mM EDTA and 0.5% bovine serum albumin). T cell analysis was performed using a CytoFlex flow cytometer (Beckman-Coulter). 10^5^ events were collected for each sample whenever possible. Below 5 × 10^4^ total events, measurements were considered inconclusive. Data analysis was performed using the Kaluza software package (Beckman Coulter). Compensation was determined using OneComp beads (Thermo). Gates were set utilizing fluorescence-minus-one controls and negative control samples. Gating was performed as follows: In a forward scatter/side scatter dot plot, a cell gate was set. These cells were further gated into viable CD3^+^ cells, which were then differentiated on a CD4/CD8 dot plot. CD4^+^ cells were analyzed for tumor necrosis factor-alpha (TNF-α) and CD154 expression as activation markers, with the fraction of double-positive cells constituting the spike-activated CD4^+^ T-cells. CD8^+^ cells were analogously analyzed for interferon-gamma (IFN-γ) and TNF-α expression, with the fraction of double-positive cells constituting the spike-activated CD8^+^ T-cells. Measurements were baseline-corrected by subtracting the fraction of double-positive cells observed in the negative controls. The threshold for activated CD4^+^ and CD8^+^ T-cells was established at 0.03%, considering a baseline level below 0.01%.

#### Anti-nucleocapsid-IgG enzyme-linked immunosorbent assay (ELISA)

Frozen plasma samples were thawed on ice and diluted 1:1000 (in two steps of 1:50 and 1:20) in Assay Buffer B as recommended by the manufacturer. Standard dilutions of an anti-N-IgG antibody were included in duplicates. All further steps were performed as outlined in the manufacturer's protocol. Absorbance was measured at 450 nm and 570 nm in a SpectraMax ABS Plus ELISA plate reader (Molecular Devices).

### Quantification and statistical analysis

Statistical differences between two experimental groups were calculated using the two-tailed Student’s *t*-test. Statistical differences between three or more groups were calculated using the one-way ANOVA test. A significance level of *p* < 0.05 was applied throughout the study. Calculations were performed with GraphPadPrism (ANOVA, *t*-tests) or SPSS (Fisher’s exact test). For the N-directed Ab ELISA analysis, a 4-parameter logistic regression was performed to fit the standard curve and calculate the sample concentrations, using R packages “drc” and “dplyr” and Rstudio software.
