# Cell-based immune anticipation of the omicron variant in SARS-CoV-2 triple-vaccinated cancer patients

**DOI:** 10.1016/j.isci.2025.113890

**Published:** 2025-11-04

**Authors:** Mario Mairhofer, Lea Kausche, Sabine Kaltenbrunner, Maria Pammer, Riad Ghanem, Maike Stegemann, Clemens A. Schmitt

## Main text

(iScience *28*, 113727; November 21, 2025)

During copyediting, the production team erroneously changed the title from “Cell-based immune anticipation of the Omicron variant in SARS-CoV-2 triple-vaccinated cancer patients” to “Cell-based immune anticipation of the omicron variant in patients with SARS-CoV-2 triple-vaccinated cancer.” The title has now been corrected online. Production apologizes for any inconvenience and confusion caused.

