# Supplementary material for: Cell-based immune anticipation of the omicron variant in SARS-CoV-2 triple-vaccinated cancer patients
Source: iScience. 2025 Oct 9;28(11):113727. doi: 10.1016/j.isci.2025.113727 (PMC12589992; doi:10.1016/j.isci.2025.113727)
Supplement: Document S1. Figures S1 and S2 and Tables S1–S4 [file mmc1.pdf]

iScience, Volume *28*

Supplemental information

Cell-based immune anticipation of the omicron variant in SARS-CoV-2 triple-vaccinated cancer patients

Mario Mairhofer, Lea Kausche, Sabine Kaltenbrunner, Maria Pammer, Riad Ghanem, Maike Stegemann, and Clemens A. Schmitt

Supplementary Figure 1:

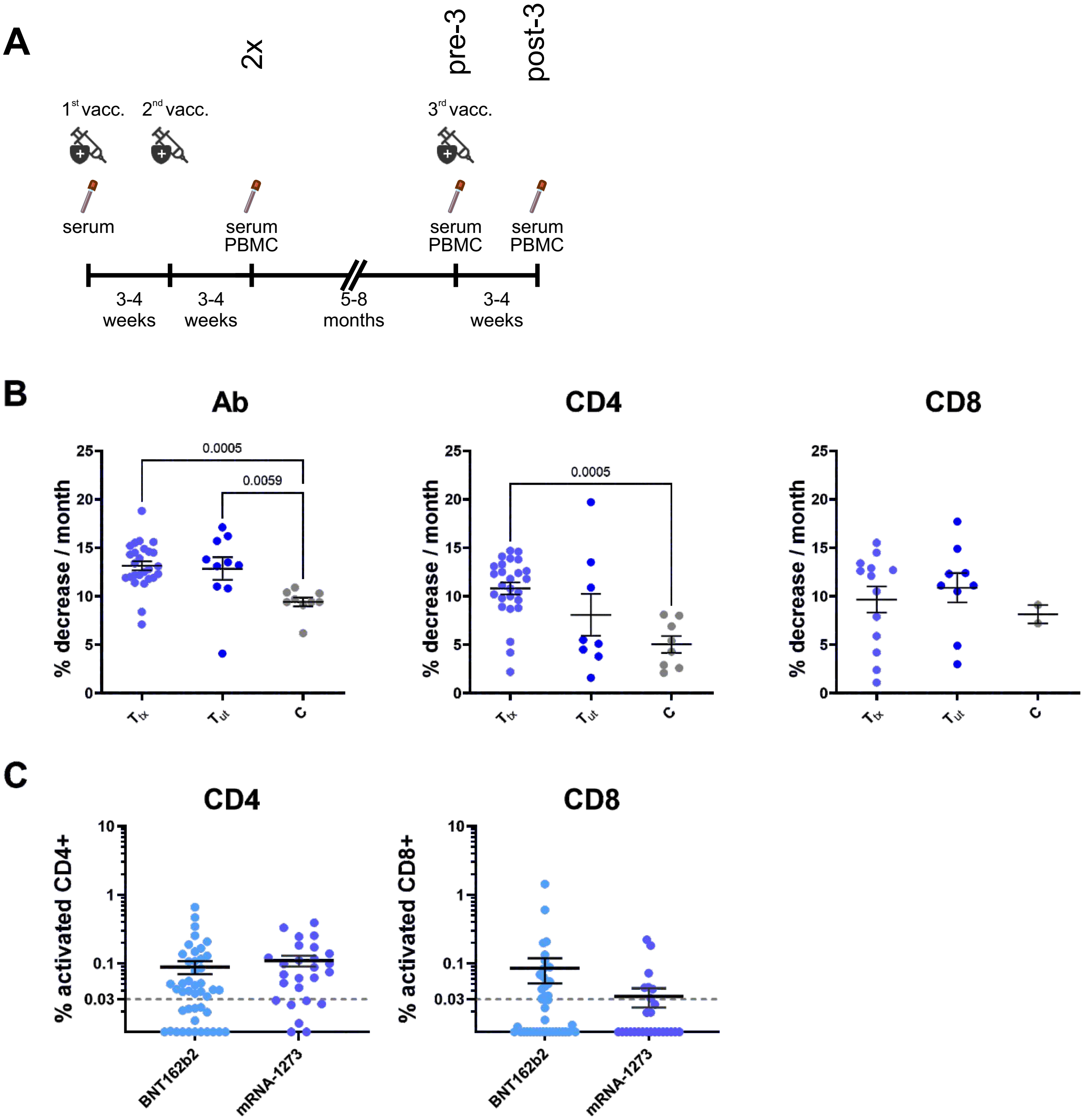

Suppl. Figure 1 | Additional information on response waning in cancer patients.
(A) Schematic of the time-line of basal double-vaccination followed by the '2x', pre-boost - immediately prior to the third vaccination - and post-boost time-points to collect peripheral blood from the participants. (B) Relative monthly decline of the indicated humoral and cellular response parameters according to a linear mathematical approximation model in the indicated sub-cohorts. Error bars indicate mean ± SEM, and numbers above the braces indicate the p values of the given comparison (with $p < 0.05$ considered significant). (C) Pre-boost analyses of spike-specific activated CD4+ (left) and CD8+ T-cells of 45 participants that received a BNT162b2-based (left) and 26 participants that received a mRNA-1273-based basal double-vaccination (corresponds to Fig. 1D).

Supplementary Figure 2:

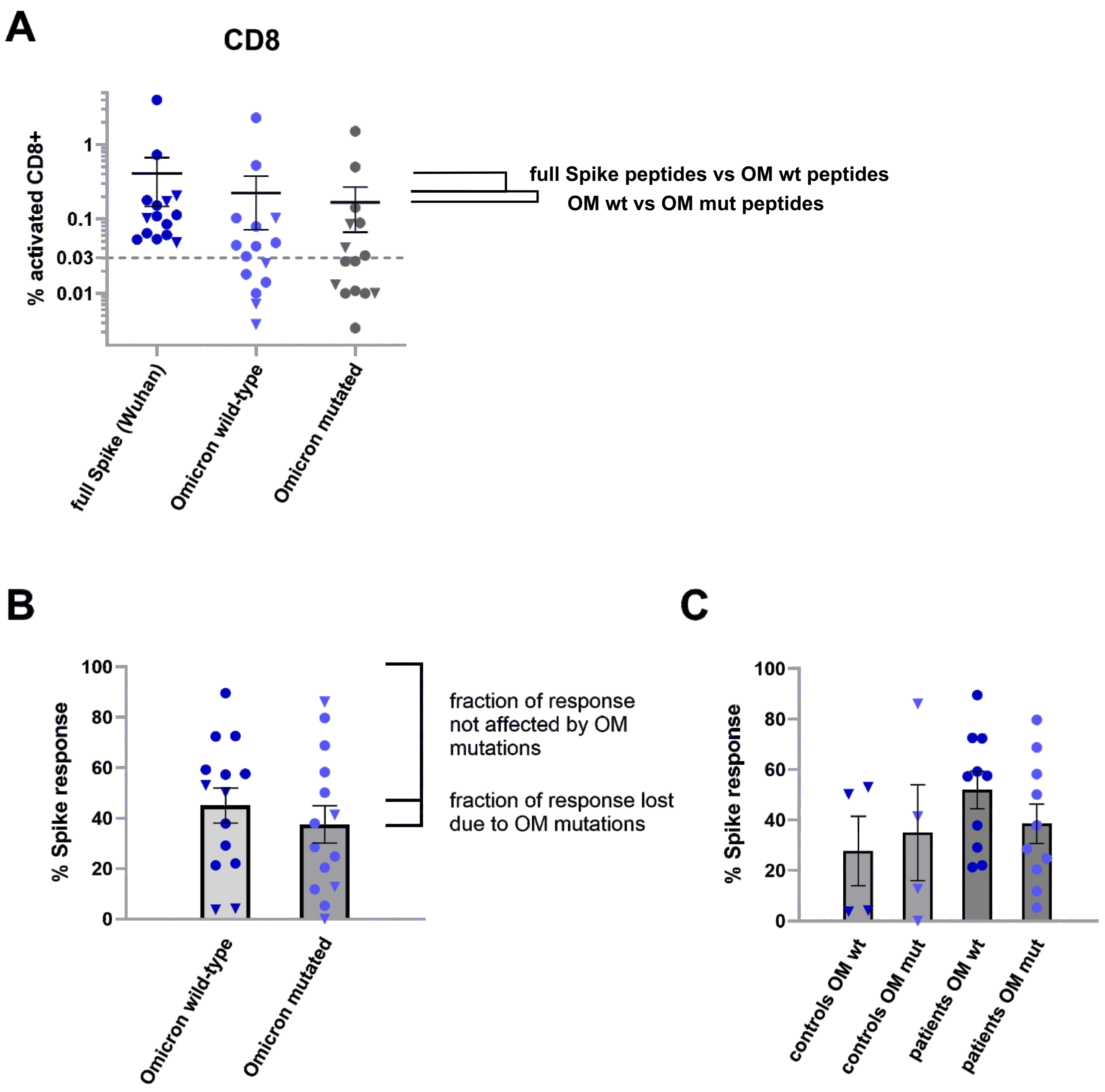

Suppl. Figure 2 | Anticipation of Omicron (OM) reactivity in CD8+ T-cells. (A) PBMC of participants (n = 14) with a strong CD8+ T-cell immune response prior to the booster vaccination were stimulated either with the full spike peptide complement (left), or with only the peptides potentially mutated in the OM variant, either with their wild-type (middle) or mutated sequences (right). Activated CD8+ T-cells are graphed. Control samples are indicated by triangles throughout the figure. (B) The OM wild-type and mutated measurements are normalized to the full spike peptide responses, visualizing which fraction of the response is lost either due to the reduced peptide complement or specifically due to the numerous OM mutations. (C) The normalized measurements were further split into controls (n = 4) and patients (n = 10) to reveal potential differences. No statistically significant differences were observed, but data highlight individual variability of OM wild-type and mutated peptide reactivity.

Suppl. Table S1. Results & statistical parameters Figure 1 & Supplementary Figure 1

Figure 1A[1]:

		mean	SEM	95% C.I.	n	p (paired t-test)
**AB titer**	T_{tx} 2x	2.031	401	(1206-2856)	27	<0.0001
	T_{tx} pre-3	360	108	(139-582)		
	T_{ut} 2x	1.722	684	(175-3268)	10	0.0540
	T_{ut} pre-3	385	193	(-52-823)		
	C 2x	998	173	(599-1398)	9	0.0005
	C pre-3	211	48	(99-322)		
**activated $CD4^+$ T cells**	T_{tx} 2x	0.3043	0.0474	(0.2072 - 0.4013)	29	<0.0001
	T_{tx} pre-3	0.0976	0.0159	(0.0650 - 0.1301)		
	T_{ut} 2x	0.5388	0.1557	(0.1797 - 0.8980)	9	0.0355
	T_{ut} pre-3	0.2083	0.0529	(0.0862 - 0.3303)		
	C 2x	0.1445	0.0304	(0.0744 - 0.2146)	9	0.0916
	C pre-3	0.0836	0.0129	(0.0538 - 0.1134)		
**activated $CD8^+$ T cells**	T_{tx} 2x	0.2098	0.0584	(0.0846 - 0.3349)	15	0.0083
	T_{tx} pre-3	0.0590	0.0151	(0.0265 - 0.0914)		
	T_{ut} 2x	0.1276	0.0663	(-0.0253 - 0.2805)	9	0.0909
	T_{ut} pre-3	0.0398	0.0216	(-0.0100 - 0.0897)		
	C 2x	0.0912	0.0072	(0.0710 - 0.1111)	5	0.9073
	C pre-3	0.0876	0.0288	(0.0078 - 0.1675)		

Figure 1B:

		mean	SEM	95% C.I.	n	p (Anova)	Multiple comparisons		
**AB % primary response**	T_{tx}	16.0	2.5	(10.9 - 21.0)	26	0.4297			
	T_{ut}	21.6	6.2	(7.6 - 35.5)	10				
	C	21.4	3.2	(14.0 - 28.7)	9				
**$CD4^+$ % primary response**	T_{tx}	32.1	3.7	(24.5 - 39.8)	26	0.0118	0.0298	0.7343	0.0108
	T_{ut}	52.6	10.9	(26.7 - 78.4)	8				
	C	56.4	8.3	(36.8 - 76.0)	8				
**$CD8^+$ % primary response**	T_{tx}	36.4	8.5	(17.8 - 54.9)	13	0.8671			
	T_{ut}	32.2	8.3	(13.1 - 51.3)	9				
	C	26.4	6.5	(-56.2 - 109)	2				

Figure 1C:

		mean	SEM	95% C.I.	n	p (Anova)	Multiple comparisons		
**Time to booster vaccinatio**	T_{tx}	6.4	0.13	(6.1 - 6.6)	40	<0.0001	0.6850	<0.000	<0.0001
(2x to pre-3, in months)	T_{ut}	6.3	0.23	(5.8 - 6.8)	12				
	C	8.4	0.24	(7.8 - 8.9)	9				

Figure 1D:

		mean	SEM	95% C.I.	n	p (t-test)
**AB titer[2]**	BNT162b2	200	35	(30 - 171)	45	0.0010
	mRNA-1273	447	119	(202 - 692)	26	

SupplFig 1B:

		mean	SEM	95% C.I.	n	p (Anova)	Multiple comparisons		
**AB % decrease/month**	T_{tx}	13.2	0.5	(12.2 - 14.1)	26	0.0019	0.7584	0.0059	0.0005
	T_{ut}	12.9	1.2	(10.2 - 15.5)	10				
	C	9.4	0.5	(8.4 - 10.5)	9				
**$CD4^+$ % decrease/month**	T_{tx}	10.8	0.6	(9.5 - 12.1)	26	0.0016	0.0799	0.1136	0.0005
	T_{ut}	8.1	2.2	(2.9 - 13.2)	8				
	C	5.0	0.9	(3.0 - 7.1)	8				
**$CD8^+$ % decrease/month**	T_{tx}	9.7	1.3	(6.7 - 12.6)	13	0.7036			
	T_{ut}	10.9	1.5	(7.4 - 14.4)	9				
	C	8.2	1.0	(-3.9 - 20.2)	2				

SupplFig 1C:

		mean	SEM	95% C.I.	n	p (t-test)
**activated $CD4^+$ T cells[2]**	BNT162b2	0.0889	0.0192	(0.0503 - 0.1276)	45	0.4769
	mRNA-1273	0.1099	0.0197	(0.0694 - 0.1504)	26	
**activated $CD8^+$ T cells[2]**	BNT162b2	0.0853	0.0340	(0.0169 - 0.1537)	45	0.2560
	mRNA-1273	0.0332	0.0103	(0.0120 - 0.0544)	26	

[1] The 2x responses depicted in Figure 2A and analysed here represent a subset of the post-vaccine responses presented in Mairhofer et al., Cancer Cell 2021.

[2] Three newly recruited patients with cancer had received basic (2x) immunization with an Adenoviral vaccine and were excluded from the analysis.

Suppl. Table S2. Results & statistical parameters Figure 2

Figure 2A:

		mean	SEM	95% C.I.	n	p (paired t-test)	failure rate (%)
**AB titer**	T_{tx} pre-3	199	54	(92 - 306)	59	<0.0001	32.2
	T_{tx} post-3	2.775	321	(2133 - 3418)			18.6
	T_{ut} pre-3	297	134	(9 - 585)	15	<0.0001	13.3
	T_{ut} post--3	3.157	555	(1966 - 4348)			0
	C pre-3	179	40	(91 - 267)	12	<0.0001	0
	C post-3	4.344	537	(3161 - 5527)			0
**activated CD4$^+$ T cells**	T_{tx} pre-3	0.0705	0.0094	(0.0516 - 0.0894)	59	0.0061	37.3
	T_{tx} post-3	0.3065	0.0832	(0.1399 - 0.4731)			11.9
	T_{ut} pre-3	0.1894	0.0498	(0.0826 - 0.2963)	15	0.0053	20
	T_{ut} post--3	0.5826	0.1478	(0.2657 - 0.8996)			0
	C pre-3	0.0790	0.0123	(0.0521 - 0.1060)	12	0.1685	8.3
	C post-3	0.1110	0.0211	(0.0645 - 0.1573)			8.3
**activated CD8$^+$ T cells**	T_{tx} pre-3	0.0456	0.0112	(0.0216 - 0.0663)	59	0.1051	62.7
	T_{tx} post-3	0.1168	0.0800	(0.0103 - 0.3304)			57.6
	T_{ut} pre-3	0.1444	0.0940	(-0.0572 - 0.3460)	15	0.2947	53.3
	T_{ut} post--3	0.7057	0.6084	(-0.5991 - 2.011)			26.7
	C pre-3	0.0440	0.0708	(0.0111 - 0.0800)	12	0.2825	58.3
	C post-3	0.1703	0.0157	(-0.0389 - 0.2726)			58.3

Figure 2D:

		mean	SEM	95% C.I.	n	p (Anova - multiple comparisons)	failure rate (%)
**AB titer**	control	4.344	538	(3161 - 5527)	12	control vs NHL p<0.0001	0
	solid	4.434	331	(3759 - 5109)	31	solid vs NHL p<0.0001	0
	NHL	863	327	(183 - 1542)	22		40.9
	MPN	2.979	588	(1698 - 4260)	13	MPN vs NHL p=0.0019	15.4
	MM	1.992	882	(-93 - 4077)	8		0
**activated CD4$^+$ T cells**	control	0.1109	0.0211	(0.0642 - 0.1573)	12	control vs MM p=0.0058	8.3
	solid	0.2563	0.0532	(0.1476 - 0.3649)	31	solid vs MM p=0.0104	3.2
	NHL	0.4224	0.1070	(0.1998 - 0.6449)	22		9.1
	MPN	0.2128	0.0644	(0.0724 - 0.3531)	13	MPN vs MM p=0.0151	23.1
	MM	0.8523	0.5669	(-0.4881 - 2.193)	8		12.5
**activated CD8$^+$ T cells**	control	0.1168	0.0708	(-0.0389 - 0.2726)	12		58.3
	solid	0.2075	0.1285	(-0.0549 - 0.4699)	31		45.2
	NHL	0.5005	0.4168	(-0.3662 - 1.367)	22		63.6
	MPN	0.2199	0.1910	(-0.1963 - 0.6360)	13		46.2
	MM	0.0417	0.0120	(0.0134 - 0.0699)	8		50

Suppl. Table S3. Results & statistical parameters Figure 3

**Figure 3A:**						
		**positivity rate (%)**				
**pre-3**	$CD4^+$	17.4				
	$CD8^+$	10.5				
**post-3**	$CD4^+$	32.6				
	$CD8^+$	11.6				
**Figure 3B:**						
		**mean**	**SEM**	**95% C.I.**	**n**	**p (paired t-test)**
**activated $CD4^+$ T cells M/N**	pre-3	0.1452	0.0577	(0.0215 - 0.2690)	15	0.6571
	post-3	0.1698	0.0637	(0.0332 - 0.3064)		
**activated $CD4^+$ T cells Spike**	pre-3	0.1718	0.0423	(0.0814 - 0.2623)	15	0.0493
	post-3	0.4184	0.1510	(0.0946 - 0.7423)		
**Figure 3C:**						
		**mean**	**SEM**	**95% C.I.**	**n**	**p (paired t-test)**
**anti-N Ab ELISA**	pre-3	13.94	2.30	(9.25 - 18.64)	33	0.6307
(ng/mL)	post-3	14.63	2.21	(10.14 - 19.13)		
**Figure 3D:**						
**pre-3:**		**mean**	**SEM**	**95% C.I.**	**n**	**p (t-test)**
AB titer	INF+VACC	271	102	(58 - 483)	21	0.4592
	VACC	195	48	(99 - 290)	65	
activated $CD4^+$ T cells	INF+VACC	0.1380	0.0325	(0.0702 - 0.2058)	21	0.0443
	VACC	0.0786	0.0130	(0.0525 - 0.1047)	65	
activated $CD8^+$ T cells	INF+VACC	0.1657	0.0706	(0.0184 - 0.3130)	21	0.0009
	VACC	0.0281	0.0032	(0.0216 - 0.0345)	65	
**post-3:**		**mean**	**SEM**	**95% C.I.**	**n**	**p (t-test)**
AB titer	INF+VACC 3x	3.388	359	(2661 - 4115)	40	0.2353
	VACC	2.776	362	(2047 - 3504)	46	
activated $CD4^+$ T cells	INF+VACC 3x	0.3629	0.0693	(0.2228 - 0.5030)	40	0.6073
	VACC	0.2964	0.1039	(0.0872 - 0.5057)	46	
activated $CD8^+$ T cells	INF+VACC 3x	0.5102	0.2520	(0.0003 - 1.020)	40	0.0465
	VACC	0.0355	0.0053	(0.0247 - 0.0462)	46	

Suppl. Table S4. Quantitative results & statistical parameters Figure 4 & Suppl. Figure 2

Figure 4A:

		mean	SEM	95% C.I.	n	p (Anova)	Multiple comparisons	
**activated CD4$^+$ T cells**	full Spike (Wuhan)	0.2124	0.0484	(0.1116 - 0.3131)			] 0.0038	] 0.0002
	Omicron wild-type	0.0803	0.0216	(0.0354 - 0.1252)	22	0.0006	] 0.3674	
	Omicron mutated	0.0404	0.0095	(0.0206 - 0.0601)				

Figure 4B:

		mean	SEM	95% C.I.	n	p (paired t-test)
**% Spike response**	Omicron wild-type	37.7	4.0	(29.3 - 46.0)	22	<0.0001
	Omicron mutated	22.5	4.4	(13.4 - 31.5)		

Figure 4C:

		mean	SEM	95% C.I.	n	p (Anova)
**% Spike response**	controls OM wt	29.8	5.1	(18.1 - 41.5)	9	0.0392
	controls OM mut	21.7	5.8	(8.3 - 35.1)	9	
	patients OM wt	43.1	5.4	(29.7 - 50.0)	13	
	patients OM mut	23.0	6.3	(11.9 - 32.8)	13	

Supplementary Figure 2A:

		mean	SEM	95% C.I.	n	p (Anova)
**activated CD8$^+$ T cells**	full Spike (Wuhan)	0.5264	0.3537	(-0.2617 - 1.315)		
	Omicron wild-type	0.2912	0.2060	(-0.1679 - 0.7503)	11	0.6574
	Omicron mutated	0.2143	0.1375	(-0.0920 - 0.5206)		

Supplementary Figure 4B:

		mean	SEM	95% C.I.	n	p (paired t-test)
**% Spike response**	Omicron wild-type	44.6	8.5	(25.7 - 63.6)	11	0.4052
	Omicron mutated	38.2	7.9	(20.6 - 55.8)		

Supplementary Figure 4C:

		mean	SEM	95% C.I.	n	p (Anova)
**% Spike response**	controls OM wt	27.8	13.8	(-16.1 - 71.6)	4	0.4309
	controls OM mut	35.0	19.1	(-25.7 - 95.7)	4	
	patients OM wt	54.3	9.6	(30.7 - 77.9)	7	
	patients OM mut	40.0	7.6	(21.4 - 58.6)	7	
